# Implementation of 3D Optical Scanning Technology for Automotive Applications

**DOI:** 10.3390/s90301967

**Published:** 2009-03-17

**Authors:** Abdil Kuş

**Affiliations:** Uludağ University, Vocational School of Technical Sciences, 16059 Görükle Campus, Bursa – Turkey; E-Mail: abdilkus@uludag.edu.tr

**Keywords:** 3D optical scanning, reverse engineering, part inspection, 3D modeling

## Abstract

Reverse engineering (RE) is a powerful tool for generating a CAD model from the 3D scan data of a physical part that lacks documentation or has changed from the original CAD design of the part. The process of digitizing a part and creating a CAD model from 3D scan data is less time consuming and provides greater accuracy than manually measuring the part and designing the part from scratch in CAD. 3D optical scanning technology is one of the measurement methods which have evolved over the last few years and it is used in a wide range of areas from industrial applications to art and cultural heritage. It is also used extensively in the automotive industry for applications such as part inspections, scanning of tools without CAD definition, scanning the casting for definition of the stock (i.e. the amount of material to be removed from the surface of the castings) model for CAM programs and reverse engineering. In this study two scanning experiments of automotive applications are illustrated. The first one examines the processes from scanning to re-manufacturing the damaged sheet metal cutting die, using a 3D scanning technique and the second study compares the scanned point clouds data to 3D CAD data for inspection purposes. Furthermore, the deviations of the part holes are determined by using different lenses and scanning parameters.

## 1. Introduction

Reverse engineering (RE) is a process of building from an existing physical object an identical 3D-CAD model, which can be used for manufacturing or other applications. An example application is where CAD data is not available, unusable, or insufficient for exiting parts that must be duplicated or modified. One of other practical applications is tool and die-making in automotive industry [1–3].

Technological developments have resulted in important changes in design and manufacturing methods in the automotive industry. Customers not only expect higher quality, lower price and higher performance, but they also require the earliest delivery of products. Meeting these requirements is almost impossible without computer based design and production technologies [4–6]. 3D CAD modeling of objects by hand which have not pre-existing computer models is extremely time consuming and difficult with even the advanced 3D software packages. To aid in the modeling process, 3D scanners are used to capture the object shape and generate a high resolution model of the object. Optical scanning systems are one of the widely used 3D scanning systems in a wide range of areas such as automotive industry, medical applications, architectural and historical preservation. 3D Optical scanning systems can capture millions of points in a second to create point clouds data. The resulting 3D data can then be transferred to a CAD system for 3D surface or solid modeling, finite element analysis, tool design and tool path generation.

**Figure 1.**
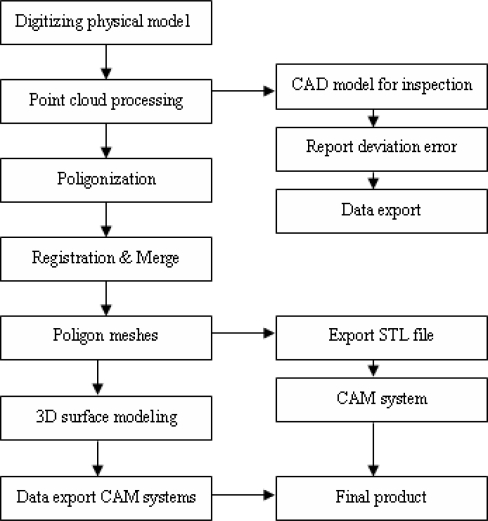
3D Digitizing process and it’s applications in the automotive industry.

Today in the automotive industry, 3D scanning is used in many different fields (Figure 1). Some examples of these typical applications are:

1. 3D-optical scanning systems which can use to obtain geometrical data where technical drawings or 3D CAD data of the parts are not available.
2. Inspection and Quality control.
3. Reducing production time by minimizing the non-machining time of CNC machines by identifying STL data obtained from scanning of casting parts as stock model to CAM software.
4. Reverse engineering and rapid prototyping.

In this study, two examples are presented showing the use of 3D optical scanning system in the automotive industry. The both examples included various steps, ranging from the 3D optical digitization of the damaged die surfaces and sheet metal part produced out of this die to the multi-view registration of the views, the generation of the polygonal models, the generation of the 3D CAD models and tool path generation for the CNC machine tools.

## 2. 3D Scanning Technology

### 2.1. An Overview of 3D Scanning Technology

3D scanning technologies are potential tools for increasing productivity, while at the same time securing quality in product development. Generally, 3D scanning can be of big help in resolving the issues concerning ways of creating 3D CAD data for objects that do not have pre-existing computer models. Creating good digital representations is often of crucial importance when using today’s manufacturing methods.

Today 3D scanners are available to digitize objects from microscopic to large constructions in size. Data points are captured with speeds ranging from a few points per second to more than a million points per second. There are handheld manual devices available as well as large size automatic scanning equipment [1].

There are mainly two methods for obtaining coordinates of an object’s geometrical shape. The first one is mechanical method which uses mechanical arms where the object is fixed on a table; the coordinates of the points picked by the inspector by means of touch-probes are transferred to the computer. With this system, measurement of formed and large surfaces may take hours or even days depending on the details of the object and accuracy of the measurement required. Accuracy levels up to 1 μm can be achieved by using this method. This level of sensitivity depends on the experience of the inspector and type of the equipment used. The second one is non-contacting scanning methods which can be classified in to three main categories optical, acoustic and magnetic.

Optical scanning systems based on techniques such as laser scanning, fringe projection, photogrammetry etc. are being applied successfully for the 3D measurement and virtual reconstruction of object surfaces in many areas. Fringe projection scanning systems generally work with white structured light where the light pattern is projected on the object’s surface while one or two cameras record the reflected light while laser scanning systems can obtain data by sending laser light onto the object and processing the data obtained from the returning light [7–9]. The advantages of these scanners are that they are more portable compared to contact systems and their sensitivity levels are partially independent of the inspector.

Optical scanning systems, e.g. laser or fringe projection can obtain a large amount of point data in a short period of time and the accuracy of laser systems vary typically from 1 μm up to 20 μm, whereas fringe projection systems have the capability of 10 μm up to 60 μm. Since the accuracy of the non contact systems are continually improving, they are now widely adopted for many applications in the industry [10].

Optical technology is generally preferred method because it gives a greater flexibility in the digitization of surfaces and provides higher resolution and accuracy when compared to mechanical technology [11,12]. Because of speed of measurement and greater flexibility, there is an increasing demand for optical scanning systems [13–15]. The advantage of contacting devices is that they do not depend on the color and reflective characteristics of the surfaces to be scanned which might be the case with optical scanners.

### 2.2. The 3D scanner used in this study

**Figure 2.**
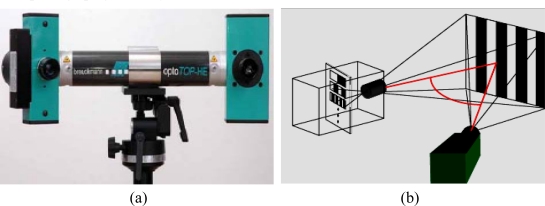
Breuckmann Opto-TOP digitizing system: (a) image of the instrument; and (b) Setup fringe projection system

The Opto-TOP HE Breuckmann 3D optical scanning system used in this study utilizes 3D white light fringe projection technology to provide a fast and extensive capture of complex surfaces. This allows the simultaneous measurement of a large portion of the object in a single view. Over a million points can be captured in each image. Each of these scanned images takes around one second (Figure 2). The equipment is portable and does not require the use of a mechanical positioning device.

The scanner is designed to allow a quick change of lenses. The projector which is connected to the camera by means of a carbon fiber bar, projects the fringe patterns in rapid sequences providing a flexible system capable of measuring very small or very large objects. A carbon fiber base structure ensures optimum mechanical and thermal stability of the sensor. Calibration may be performed by the user within minutes, ensuring a high degree of accuracy. Featuring 1.4 mega pixels (optionally 6.6 mega pixels) and a digital zoom, the digital camera provides maximum resolution [16].

## 3. Case Study 1: 3D scanning of the Die

This study was carried out at the Tunaoglu Company in collaboration with University of Uludag, Turkey. The company required 3D CAD models of the damaged surfaces of a set of dies consisting of forming, cutting and punching operations used for the production of car clutch housings. Students and staff from the University visited the company to investigate the requirements as part of the University-Company support and cooperation procedure. The company initially investigated the potential use of a mechanical contact method for the creation of the die. But this option was not considered as the estimated time was excessive and the required expertise was not available locally. Engineers from the company informed the academic team that the damaged tools were producing a high percentage of defect parts and a new set of tools was needed. The procedure for the scanning process was as follows:

1. Calibration of the sensors
2. Preparation of the scanned part
3. Implementing the scanning process
4. Cleaning noise data
5. Merging images
6. Translating to STL format
7. 3D CAD Modeling

### 3.1. Calibration of the sensor

In the automotive industry, part tolerances depend on the location and function of the parts in the whole body. During the scanning process, different lenses and bar combinations are used to achieve the tolerances required.

**Figure 3.**
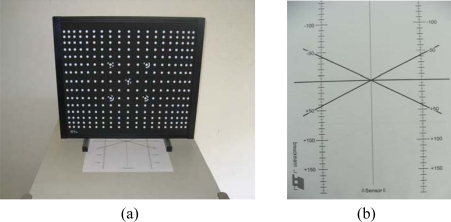
(a) Calibration plate and (b) Angle master

Calibration is an essential part of setting up and operating a position measuring device. Systematic sensing errors can occur through lens distortions, non-linear electronics in cameras, and similar sources. The system was calibrated before scanning of the die surfaces commenced. System correction was then performed using lens modification. This correction was made by measuring the calibration plates from different distances and angles (Figure 3).

### 3.2. Preparation of the scanned part

**Figure 4.**
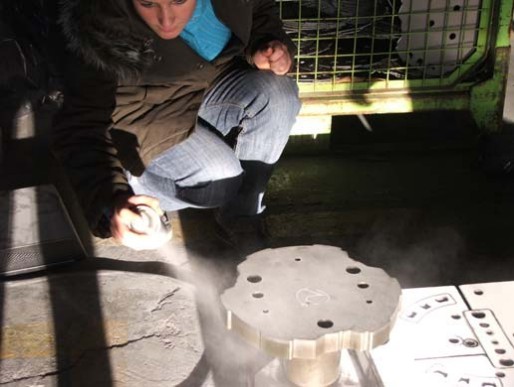
Preparation of the scanned part.

The scanned die was used to perform the forming, cutting and punching operations. Before the scanning process, preliminary preparation was carried out by using a calcite spray (Figure 4). Calcite spray prevents the shiny reflections from the surfaces in order to achieve a better quality scanning. The thickness of this spray is between 7–10 μm, but due to its characteristic the thickness of the powder never exceeds a 20 μms level. Lighting conditions also affect the scanning process. For the final measurements two different scanning in two different lighting conditions were carried out in the factory. During scanning 460 mm × 610 mm × 440 mm image taking lenses were used.

### 3.3. Scanning and Processing

**Figure 5.**
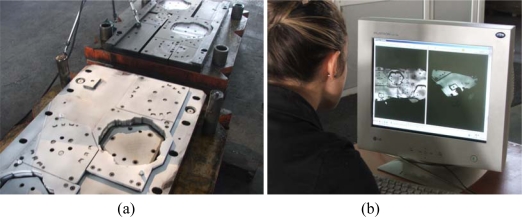
(a) Scanning male and female dies, (b) merging images.

The scanned die part was steel with the dimensions of 1000×1500×250 millimeters and a very complex object (Figure 5).

To create the CAD model the contours were needed for the male and female dies. In this scanning study different images were taken; each process took around one second with a tolerance of 60 μm (Figure 5a). After merging the images the scanning system enabled an accuracy of approximately 20 μm over the tool length.

The male die was scanned using 35 different positions which took around 35 minutes which is given with details in (Table 1). For the female die 19 different scans were taken. Each scan data (shots) were combined using specific geometries on the die surfaces (Figure 5b).

The basic combining procedure requires the selection of at least three fiduciary points in each of the views. There were overlap regions of measurements during the merging process. Overlapping points were deleted and triangle polygons were created.

### 3.4. Generation of a 3D CAD model from scanning data

**Figure 6.**
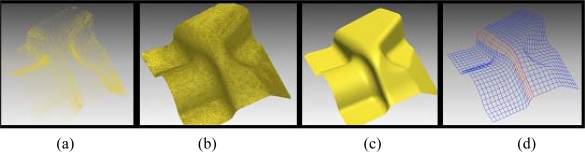
Surface modeling procedure from scanned data: a) Point cloud, b) polygon meshes, c) mesh treatment, d) surface modeling.

The data generated during 3D-scanning, i.e. the digital points cloud data in *X*, *Y*, *Z* coordinates, is exported to a model reconstruction reverse engineering system software to be transformed in a conceptual model supported by a triangular surface geometry or by a CAD-surface data (Figure 6).

In reverse engineering software there are many operations to improve the scanned point cloud data. Noisy data can be improved and the size of the data set reduced (Figure 6a). Triangulation process begins after improving the point data set (Figure 6b). In mesh treatment, the process is specific to working with the polygonal mesh model, such as cleaning abnormal polygon meshes, redefining the surface by smoothing, re-meshing, or decimation and to prepare the polygonal model for rapid prototyping or NURBS surfacing (Figures 6c–d).

**Figure 7.**
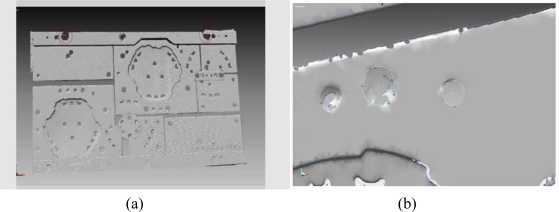
(a) STL file after combining scan shots, (b) Determining of the hole contours.

In this case study, the polygon data was recorded as an STL file and then transferred to RapidForm software to process and construct the die. These triangular representations of the 3D surface geometry data were decimated for reducing the file size. Location and position of the holes and the shape of the cutting contours were paramount for the correct operation of the die in Figure 7.

**Figure 8.**
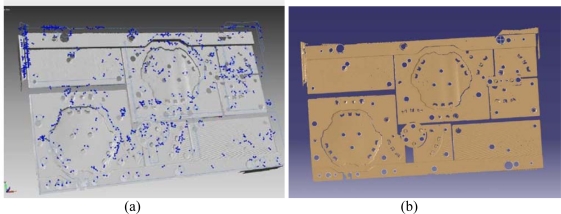
(a) Using silhouette function for contours, (b) 3D cad model of the die.

The following steps were followed to obtain an accurate 3D CAD model:

1. Coordinate system of the scanning data was determined using a vector that was generated from a marked hole centre on the die. Then a plane was created using die upper surface.
2. Using the reverse engineering Rapidform software, exact locations of the holes were identified and the hole diameters were measured using the curve module silhouette tool. This data was then transferred to TopSolid CAD-CAM package.
3. Separate border curves and circles were obtained on the die for solid modeling (Figure 8.a).
4. Created solid model with obtained curves by using cad functions (Figure 8.b)
5. Since the constructed die had progressive characteristics, distances between the stations on the die was processed separately and faults on the scanned die were determined.
6. CNC tool path and tool path simulations were successfully generated.

### 3.5. The reasons why 3D scanning technology is necessary

Contact scanning Coordinate Measuring Machines (CMMs) are widely used in the creation of the surface models in many industries. As already mentioned, however, the primary disadvantage of this approach is the relatively longer measuring times needed to complete the process. On the other hand, the use of 3D optical scanning allows the collection of large amounts of dimensional data in reasonably shorter times.

3D CAD definitions of products or dies may not be available, not be up-to-date, or may not be achievable for many reasons (i.e. very old dies, bankrupt suppliers, lost data, etc). This is when 3D scanning technologies are indispensible for creating the required 3D definitions of the product models, samples or damaged die surfaces, etc.

3D optical scanning systems can also be used by companies where a new 3D CAD system is introduced. All existing products must be modeled in order to have a fully digital archive. This 3D data then can be directly utilized in further CAD/CAM/CAE applications. These numerical applications enable product optimizations to improve the final product quality and, to increase the competitiveness of the products.

## 4. Case Study 2: Inspection of sheet-metal parts using 3D scanning

This second case study demonstrated that the 3D optical scanning system can be easily and effectively utilized for inspection purposes in manufacturing environments. Scan data can be compared to CAD data to determine, for example, the accuracy of manufactured parts versus the original design data. In this study, mass produced sheet metal parts were inspected and quality reports generated in a very short time. This is very big advantage for use in mass production lines in automotive sector. The typical work process steps of the inspection of the parts are as follows:

1. Register scan data point cloud data with ASC format or polygonal meshes with the STL format.
2. Display the deviation between scanned point cloud and CAD data.
3. Report all information of document pre-defined format such as PDF, Excel or HTML.

The press sheet metal parts produced needed to be inspected and quality reports generated for comparison with existing CAD data. The pressed component was a cross member of a car body-in-white of around 1m length. Before the scanning process, calibration and preparation of the system was carried out with 8 different scanning shots. Total measurement time was 20 minutes. Determination of edge contour detail analysis was performed in approximately 10 minutes.

**Figure 9.**
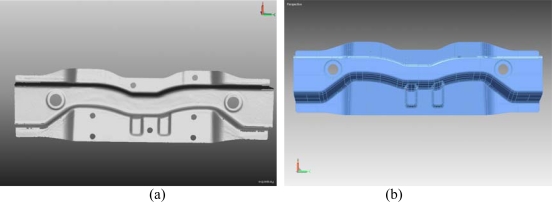
(a) STL data of scanned model, (b) 3D CAD surface model of the scanned sheet metal part.

The surface model was generated by using STL data in TopSOLID-CAD program (Figure 9). Zero datum surfaces were set by the manufacturer specification and localization was performed. Quality control report was generated using Rapidform software.

**Figure 10.**
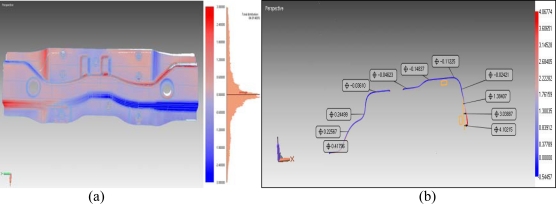
(a) Color Comparison Chart of As-Built Model to CAD surface Model, (b) The differences between scanned STL data and CAD data of a cross-section from any Y direction.

In addition, deviations on the cross-sections at y coordinates are obtained by matching STL data and CAD data obtained (Figure 10).

### 4.1. Edge Detection

**Figure 11.**
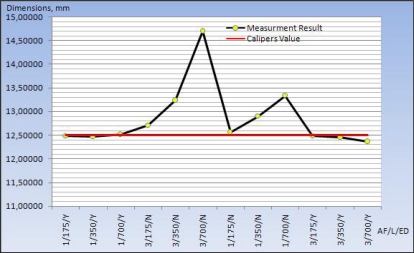
Comparison with the edge detection and caliper values using different lens and parameters for die hole diameters.

In order to show the changes an edge detection determination study was performed using different lenses and viewing areas. Measurement results of hole diameters in sheet metal part were also compared with caliper values given in (Table 3). The nearest hole diameters caliper values were obtained with 175 lenses in using different lens and parameters with edge detection in (Figure 11).

Table 3 proves the desired measurement was obtained for the centre and diameters of the holes and diameters which were within the required tolerance range for sheet-metal parts. It also shows this is effective scanning method with high accuracy as well as short measuring time.

## 5. Conclusions

3D optical scanning technology can deliver accuracy for large, complex sheet metal parts and dies used in the automotive industry. These two case studies demonstrate how multiple technologies can serve the customer’s interest by providing usable data for highly sensitive applications such as cutting and forming dies as well as sheet metal parts with very low tolerances.

In the first case study, cutting and forming dies used in production press lines were reproduced. Distances between the stations on the tool were obtained separately and faults on the scanned dies were determined. Also separate border curves and circles were obtained on the die used for the solid modeling. Desired sensitivity obtained and did not present any problem.

The second case study demonstrated that the scanning system can be easily and effectively utilized for inspection purposes in manufacturing environments. In this study mass produced sheet metal parts were inspected and quality reports generated accordingly in a very short time. This is very big advantage for use in mass production lines.

Using different lenses gives different dimensional values. Hence the importance of setting the right lens combinations to suit the required parameters and the right skill and appropriate user experience is required. Although the tolerances for panel parts in the automotive industry are generally around 0.5 mm, they are often as low as 0.1 mm. This type of scanning system can easily cope with these requirements. It is clear that there are great benefits obtained by the portability and ease of use of the system and the sensitivity level. Since the applications of 3D scanning are expected to increase dramatically in near future it will be a fundamental part of mass production automation lines in the automotive industry as well as reverse engineering applications.

## Figures and Tables

**Table 1.** Average duration of each task in scanning process and designing the 3D CAD model.

**Number of views**	35
**Scanning process**	35 min.
**Cleaning the noise**	15 min.
**Acquisition and alignment**	35 min.
**Creation/editing of the mesh**	3 h.
**Solid modeling process of the die**	6 h.

**Table 2.** Average duration of each task in scanning process and designing the sheet CAD model.

**Number of views**	8
**Scanning process**	20 min.
**Cleaning the noise**	10 min.
**Edge counter analysis**	10 min.
**Creation/editing of the mesh**	1.5 h.
**Surface modeling process of the sheet metal part**	3 h.

**Table 3.** Evaluation of hole diameters with different lenses and parameters.

	**Area of Filter**	**Lens**	**Edge of Detection**	**Camera Resolution**	**Calipers Value**	**Measurement Result**	**Difference**

**Measurement**	1	175	Yes	1,4 MP	12.5	12.48255	−0.01745
	1	350	Yes	1,4 MP	12.5	12.46718	−0.03282
	1	700	Yes	1,4 MP	12.5	12.51921	0.01921
	3	175	No	1,4 MP	12.5	12.70928	0.20928
	3	350	No	1,4 MP	12.5	13.23723	0.73723
	3	700	No	1,4 MP	12.5	14.70036	2.20036
	1	175	No	1,4 MP	12.5	12.56359	0.06359
	1	350	No	1,4 MP	12.5	12.90179	0.40179
	1	700	No	1,4 MP	12.5	13.33738	0.83738
	3	175	Yes	1,4 MP	12.5	12.48234	−0.01766
	3	350	Yes	1,4 MP	12.5	12.46513	−0.03487
	3	700	Yes	1,4 MP	12.5	12.36820	−0.13180
